# Sdf-1 (CXCL12) induces CD9 expression in stem cells engaged in muscle regeneration

**DOI:** 10.1186/s13287-015-0041-1

**Published:** 2015-03-24

**Authors:** Edyta Brzoska, Kamil Kowalski, Agnieszka Markowska-Zagrajek, Magdalena Kowalewska, Rafał Archacki, Izabela Plaskota, Władysława Stremińska, Katarzyna Jańczyk-Ilach, Maria A Ciemerych

**Affiliations:** Department of Cytology, Faculty of Biology, University of Warsaw, Miecznikowa 1, 02-096 Warsaw, Poland; Department of Molecular and Translational Oncology, Maria Skłodowska-Curie Memorial Cancer Center and Institute of Oncology, Roentgena 5, 02-781 Warsaw, Poland; Department of Immunology, Biochemistry and Nutrition, Medical University of Warsaw, Banacha 1b, 02-097 Warsaw, Poland; Departament of Systems Biology, Faculty of Biology, University of Warsaw, Pawińskiego 5a, 02-106 Warsaw, Poland

## Abstract

**Introduction:**

Understanding the mechanism of stem cell mobilization into injured skeletal muscles is a prerequisite step for the development of muscle disease therapies. Many of the currently studied stem cell types present myogenic potential; however, when introduced either into the blood stream or directly into the tissue, they are not able to efficiently engraft injured muscle. For this reason their use in therapy is still limited. Previously, we have shown that stromal-derived factor-1 (Sdf-1) caused the mobilization of endogenous (not transplanted) stem cells into injured skeletal muscle improving regeneration. Here, we demonstrate that the beneficial effect of Sdf-1 relies on the upregulation of the tetraspanin CD9 expression in stem cells.

**Methods:**

The expression pattern of adhesion proteins, including CD9, was analysed after Sdf-1 treatment during regeneration of rat skeletal muscles and mouse Pax7-/- skeletal muscles, that are characterized by the decreased number of satellite cells. Next, we examined the changes in CD9 level in satellite cells-derived myoblasts, bone marrow-derived mesenchymal stem cells, and embryonic stem cells after Sdf-1 treatment or silencing expression of CXCR4 and CXCR7. Finally, we examined the potential of stem cells to fuse with myoblasts after Sdf-1 treatment.

**Results:**

*In vivo* analyses of *Pax7-/-* mice strongly suggest that Sdf-1-mediates increase in CD9 levels also in mobilized stem cells. In the absence of CXCR4 receptor the effect of Sdf-1 on CD9 expression is blocked. Next, *in vitro* studies show that Sdf-1 increases the level of CD9 not only in satellite cell-derived myoblasts but also in bone marrow derived mesenchymal stem cells, as well as embryonic stem cells. Importantly, the Sdf-1 treated cells migrate and fuse with myoblasts more effectively.

**Conclusions:**

We suggest that Sdf-1 binding CXCR4 receptor improves skeletal muscle regeneration by upregulating expression of CD9 and thus, impacting at stem cells mobilization to the injured muscles.

## Introduction

Skeletal muscle regeneration is a complex process of tissue degeneration and reconstruction [1]. The process mostly relies on the presence of muscle-specific unipotent stem cells; that is, satellite cells. However, the myogenic potential has also been shown for other populations of stem and progenitor cells [2]. Quiescent satellite cells that express transcription factor Pax7 are located between myofiber sarcolemma and basal lamina. In the response to muscle injury these cells are activated, begin to proliferate, differentiate into myoblasts, and fuse to form multinucleated myotubes and then muscle fibres. Satellite cell-derived myoblasts start to express myogenic regulatory factors responsible for their proper differentiation, such as Myod1, Myf5, Myf6, and myogenin [3]. The satellite cells, being muscle-specific stem cells, appear to be the cells of first choice to be tested in muscle therapies [4]. Nevertheless, for many reasons, their use is still limited. Among the major obstacles preventing the application of satellite cell-derived myoblasts in therapy, one can include their restricted ability to migrate through the vasculature to effectively engraft injured muscle, their rapid cell death after transplantation, and their limited regenerative capacity after *in vitro* culture [5].

Skeletal muscles serve as a niche not only for satellite cells but also for a few other populations of stem cells. These include muscle side population cells that were identified based on their ability to exclude Hoechst 33342 dye from their cytoplasm as well as the presence of stem cell antigen Sca1 and CD45 proteins [6]. In 2002 Asakura and Rudnicki demonstrated that these cells could fuse with myoblasts *in vitro* and also contribute to the formation of 1% of new myofibres when transplanted into the damaged anterior tibialis muscle of SCID mice [7]. Next, a small population (0.25%) of muscle side population-expressing satellite cell markers (that is, Pax7 and syndecan-4) as well as side population markers (that is, ATP-binding cassette subfamily member ABCG2 transport protein and stem cell antigen Sca1) participated in the formation of 30% of muscle fibres when transplanted into a damaged mouse anterior tibialis muscle and as many as 70% of the myofibres when transplanted into the anterior tibialis muscle of mdx mice [8]. Other populations of stem cells present within the skeletal muscle are pericytes associated with small blood vessels [9], mesangioblasts [10-13], AC133 stem cells that express CD133 [14], as well as PW1^+^/Pax7^–^ interstitial cells that synthesise PW1/PEG3 protein involved in tumour necrosis factor alpha–nuclear factor-κB signalling and do not express Pax7 protein [15]. These cells could undergo myogenic differentiation *in vitro* and *in vivo*; that is, after transplantation into regenerating mouse muscles. Furthermore, various tissues, including skeletal muscle, house multipotential mesenchymal stem cells (MSCs) that are defined based on adhesion to plastic, fibroblast-like morphology, intensive proliferation *in vitro*, and the ability to differentiate into adipocytes, chondrocytes, osteoblasts, and skeletal myoblasts [16,17]. MSCs isolated from mouse bone marrow improved muscle regeneration and also reduced fibrosis; that is, excessive development of connective tissue [18]. Moreover, MSCs isolated from the synovial membrane participated in the regeneration of mouse skeletal muscles and were present in the satellite cell niche. These cells also partially improved the function of skeletal muscle of mdx mice [17]. Additionally, many experiments showed that the regeneration of skeletal muscle can also be supported by stem cells isolated from tissues other than skeletal muscle. Among these were stem cells derived from bone marrow [19], human umbilical cord blood [20,21], human umbilical cord Wharton’s jelly [22], and hematopoietic stem cells [23]. Furthermore, pluripotent stem cells such as embryonic stem cells (ESCs) [24,25] or induced pluripotent stem cells [26-28] could also follow a myogenic programme.

Even if many of the stem or progenitor cells manifest myogenic potential, they are rarely readily available for transplantation into injured muscle. First, transplantation of exogenous stem cells can be only effective when high doses of cells, ranging from 25 × 10^6^/cm^2^ to 67.5 × 10^6^/cm^2^ cells, could be administered directly into the muscle (100 injections per 1 cm^2^) [29]. Second, transplanted cells are rarely able to migrate within injured muscle and for this reason they usually remain only at the site of the injection. Next, the method of cell administration can be also an issue. Despite their myogenic potential, many of the stem cells tested were not able to engraft injured muscle when transplanted into the bloodstream. This makes their use in therapy rather difficult and limited. Presently, the major limitations that contribute to the failure of clinical trials are caused by the lack of specific methods supporting homing of the stem cells after their systemic infusion. Summarising, comprehensive *in vitro* and *in vivo* studies demonstrated that many of stem cell populations are characterised by myogenic potential; that is, the ability to differentiate into myoblasts and muscle fibres and also to colonise the satellite cell niche. Next, the transplantation of these cells could improve regeneration of damaged muscles. However, their physiological role in the reconstruction of skeletal muscle remains unexplained.

In our previous study we showed that stromal-derived factor-1 (Sdf-1, also known as CXCL12) treatment improved skeletal muscle regeneration by enhancing endogenous (not transplanted) stem cell mobilisation into injured muscle [30]. Sdf-1 belongs to the cytokine family and acts on the cells expressing receptor CXC chemokine receptor (CXCR)-4 and/or CXCR7 [31]. Moreover, we were also analysing the role of various adhesion proteins in myoblast differentiation. M-cadherin [32], adhesion protein complex composed of ADAM-12, CD9, CD81, integrin beta1, and alpha3 [33], as well as syndecan-4 were shown by us to be engaged in myoblast differentiation [34]. Next, crucial function in this process of such proteins as integrin alpha7 [35], alpha9 [36], and other adhesion proteins was shown by other studies.

In the current study, we documented how Sdf-1 impacts on myoblasts and other stem cell properties, improving their ability to participate in the skeletal muscle regeneration. We also show that preconditioning of stem cells with Sdf-1 could be an effective approach to optimise stem cell migration and engraftment to injured muscles. Our results thus underline the mechanism that could be activated in order to mobilise endogenous cells into injured tissue. Importantly, this mechanism could also be switched on in order to enhance homing of the transplanted cells to the target tissues, and thus could allow reduction of the number of cells needed for the therapy.

## Materials and methods

All procedures involving animals were approved by First Warsaw Local Ethics Committee for Animal Experimentation.

### Cell cultures

#### Rat satellite cell-derived myoblasts

Slow twitch soleus muscles were dissected from the hind limbs of 3-month-old male WAG rats. The satellite cells were isolated by muscle digestion with 0.15% pronase (Sigma-Aldrich, St. Louis, MO, United States) in HAM’s F-12 medium (Life Technologies, Carlsbad, California, United States) buffered with 10 mM HEPES (Sigma-Aldrich) supplemented with 10% foetal bovine serum (FBS; Life Technologies). Cells were plated on 2% gelatine-coated (Sigma-Aldrich) dishes in complete Dulbecco’s modified Eagle’s medium (DMEM; Life Technologies) supplemented with 10% FBS, 10% horse serum (Life Technologies), and 1% antibiotics (AB, 50 U/ml penicillin, 50 μg/ml streptomycin; Life Technologies). Cells were cultured at 37°C in an atmosphere of 5% carbon dioxide. The medium was changed every 2 days. Starting from day 3 of culture, cells were treated with 100 ng/ml Sdf-1. The control cells were cultured in the absence of Sdf-1 in the medium. The cells were subjected either to quantitative RT-PCR, immunolocalisation, or western blotting. The morphology of cells was analysed using a Nikon Eclipse TE200 microscope (Nikon Instruments, Tokyo, Japan) with Hoffman contrast.

#### C2C12 myoblasts

Mouse C2C12 myoblasts (obtained from the European Collection of Cell Cultures, Porton Down, United Kingdom) were plated in DMEM supplemented with 10% FBS and 1% AB, on 2% gelatine-coated plates. Cells were cultured at 37°C in an atmosphere of 5% carbon dioxide. The morphology of cells was analysed using a Nikon Eclipse TE200 microscope with Hoffman contrast. The cells were subjected either to Sdf-1 treatment, transfection with small interfering RNA (siRNA) complementary to mRNA encoding CXCR4, quantitative RT-PCR, or immunolocalisation or used for co-culture experiments.

#### Mouse bone marrow-derived mesenchymal stem cells

The bones were dissected from the hind limbs of 3-month-old male C57Bl6N mice carrying the lacZ transgene in the ROSA26 locus. Next, the ends of the bones were cut and bone marrow was washed out with saline using a 22 G needle. Obtained cells were washed twice with saline. Erythrocytes were then removed by gradient centrifugation in Histopaque (Sigma-Aldrich) for 20 minutes at 1,800 rpm. Obtained mononucleate cells were separated using a magnetic column (MACS; Miltenyl Biotec, Bergisch Gladbach, Germany) with anti-CXCR4 specific antibody (Abcam, Cambridge, United Kingdom), according to the manufacturer’s instruction. The CXCR4^+^ fraction of cells was cultured in α-minimum essential medium (Sigma-Aldrich) supplemented with 20% FBS, 200 mM l-glutamine (Life Technologies), and 1% AB. The morphology of cells was analysed using a Nikon Eclipse TE200 microscope with Hoffman contrast. The cells were subjected either to Sdf-1 treatment, quantitative RT-PCR, or immunolocalisation or used for co-culture experiments.

#### Mouse embryonic stem cells

ESCs constitutively expressing histone H2B-GFP were provided by Dr Kat Hadjantonakis [37]. Mitomycin-inactivated mouse embryonic fibroblasts, which served as the feeder layer for ESCs, were plated on dishes coated with 1% gelatine (Sigma-Aldrich) in DMEM supplemented with 10% FBS and 1% AB. Twenty-four hours later ESCs were seeded onto the inactivated mouse embryonic fibroblasts and cultured in knockout DMEM (Life Technologies) supplemented with 15% ES-qualified FBS (Life Technologies), 0.1 mM nonessential amino acids (Sigma-Aldrich), 200 mM l-glutamine (Life Technologies), 0.1 mM β-mercaptoethanol (Sigma-Aldrich), 1% AB, and 500 U/ml leukaemia inhibitory factor (Chemicon, Billerica, MA, United States). Prior to transfection, ESCs were separated from mouse embryonic fibroblasts by the preplating technique and cultured on dishes coated with Matrigel Matrix Growth Factor Reduced (1 mg/ml DMEM; BD Biosciences, Becton-Dickinson, San Jose, CA, United States). The morphology of cells was analysed using a Nikon Eclipse TE200 microscope with Hoffman contrast. The cells were subjected either to Sdf-1 treatment, transfection with siRNA complementary to mRNA encoding CXCR4 or CXCR7, quantitative RT-PCR, immunolocalisation, or western blotting or used for co-culture experiments.

### Sdf-1 treatment and silencing of CXCR4 or CXCR7 expression by RNA interference

C2C12 or ESCs were plated on plates covered with Matrigel Matrix Growth Factor Reduced (BD Biosciences). After reaching 30 to 40% confluence the cells were transfected with Silencer Select Pre-designed siRNA (Life Technologies) complementary to mRNAs encoding either CXCR4 9 (ID:s64091) or CXCR7 (ID:s64124). Appropriate, recommended negative control siRNA was used. siRNA duplexes were diluted in DMEM to reach the concentration of 100 pmol per plate and incubated with Lipofectamine RNAiMAX (Life Technologies), according to the manufacturer’s instructions. After 24 hours the cells were treated with Sdf-1 (10 ng/μl). Next, cells were collected 48 hours post Sdf-1 treatment and processed either for mRNA isolation followed by quantitative RT-PCR, immunolocalisation, or western blotting. The efficiency of CXCR4 or CXCR7 downregulation was tested by quantitative RT-PCR and immunocytochemistry.

### Co-culture of stem cells and mouse C2C12 myoblasts

C2C12 myoblasts were plated at density of 3 × 10^4^ in DMEM with 10% FBS and 1% AB. When C2C12 cells reached confluence and started to fuse, bone marrow-derived mesenchymal stem cells (BM-MSCs) or ESCs – control or pretreated with Sdf-1 – were seeded. Respectively, 5 × 10^6^ ESCs and 2 × 10^4^ BM-MSCs were added. After 24 hours the medium was changed for differentiation; that is, DMEM supplemented with 3% horse serum and 1% AB. After 14 days of co-culture, cells were fixed in 3% paraformaldehyde (Sigma-Aldrich) and then processed for immunolocalisation of selected antigens, as described below. Skeletal myosin heavy and light chains (Sigma-Aldrich) were localised to define differentiated myotubes in ESC and myoblast co-cultures. β-galactosidase (Abcam) was localised to identified BM-MSCs in co-cultures with myoblasts. Cell nuclei were visualised with DraQ5 (Biostatus Limited, Biostatus Ltd, Leicestershire, United Kingdom) diluted in PBS. Cultures were analysed using confocal microscope Axiovert 100 M (Zeiss, Carl Zeiss Inc., Jena, Germany) and LSM 510 application software (Carl Zeiss Inc., Jena, Germany). The same image acquisition settings were used for all comparisons. For each experimental group, the number of hybrid myotubes was counted from 50 random fields of view. Data are the mean ± standard deviation of three biological replicates. Results were analysed by Student’s *t* test using GraphPadPrism (GraphPad Software, Inc., La Jolla, CA, USA). Differences were considered statistically significant when *P* <0.05.

### Migration assay

BM-MSCs or ESCs were plated into the inserts of six-well dishes (8 μm pores; BD Biosciences). Both inserts and wells were coated with Matrigel Matrix Growth Factor Reduced. After 24 hours of culture, the medium in the lower dish was replaced with the medium supplemented with 50 ng/ml Sdf-1. Control cells were cultured in medium lacking Sdf-1. After 48 hours of culture the cells were fixed and stained with Giemsa (Merck, Merck KGaA, Darmstadt, Germany). The number of cells that migrated from the inserts and localised either at the membrane surface facing the lower dish or at the bottom of the lower dish was counted. Three independent experiments were performed for each analysis. Data are the mean ± standard deviation of three biological replicates. Statistical analysis was performed with unpaired *t* test using GraphPadPrism (GraphPad Software, Inc.). The results were considered to be significantly different when *P* <0.05.

### Muscle injury

The regeneration of slow twitch soleus skeletal muscles was induced in 3-month-old male WAG rats. Briefly, the animals were anaesthetised with pentobarbital sodium salt (Sigma-Aldrich) by an intraperitoneal injection (30 mg/kg body mass). Next, muscles were exposed, denervated, and crushed as described previously [30]. Muscles were injected with 100 ng Sdf-1 diluted in 20 μl physiological saline. Two injections, 10 μl each, were administered into two different parts of muscle. The control muscles were injected with 20 μl physiological saline. The animals were euthanised with carbon dioxide at days 1, 3, and 7 after the muscle injury. Next, injured muscles were isolated, weighed, and collected for further analysis.

### Quantitative RT-PCR

Total RNA was isolated from muscles, satellite cell-derived myoblasts, and C2C12 myoblasts using the High Pure Isolation Kit (Roche Applied Science, Penzberg, Germany), according to the manufacturer’s protocol. RNA was extracted from biological replicates (two C2C12 cultures or three primary cell cultures or three muscle samples per each experimental time point). Then 100 ng RNA from each sample was reverse-transcribed using the RT^2^ First Strand Kit (SABiosciences, Qiagen, Valencia, CA, United States) or the Transcriptor First Strand cDNA Synthesis Kit (Roche Applied Science) according to the manufacturer’s protocol, for muscles and myoblasts, respectively. Next, mRNA levels in muscles and satellite cell-derived myoblasts were examined using a custom PCR array (SABiosciences) for the *genes* m-cadherin, ADAM12, syndecan-4, CD9, CD81, integrin beta1 (itgb1), alpha3 (itga3), alpha7 (itga7), and alpha9 (itga9) according to the manufacturer’s protocol. mRNA levels in C2C12 myoblasts were examined using a custom PCR array based on Universal ProbeLibrary (Roche Applied Science) for the following *genes*: m-cadherin, ADAM12, CD9, CD81, itgb1, itga3, itga7, and itga9. Hypoxanthine phosphoribosyltransferase 1 (Hprt1) [RefSeq:NM_012583], glyceraldehyde-3-phosphate dehydrogenase (Gapdh) [RefSeq:NM_017008], beta-2-microglobulin (B2m) [RefSeq:NM_012512], and acidic ribosomal phosphoprotein P1 (Rplp1) [RefSeq:NM_001007604] were used as the candidate reference genes. Quantitative real-time PCR analyses were performed with the RT^2^ Real-Time PCR Master Mix (SABiosciences) in the 7500 Fast Real-Time PCR System (Applied Biosystems, Foster City, CA, United States) or with the LightCycler 480 Probes Master 9.0 (Roche Applied Sciences) in the LightCycler 480 (Roche Applied Sciences), according to the PCR array manufacturer’s instruction. Threshold cycle (Ct) values of the analysed amplicons were determined with SDS 2.1 software (Applied Biosystems) or LightCycler® 480 Software (Roche Applied Science). Expression levels were calculated with the 2^–(ΔCT)^ formula using DataAssist™ software (Applied Biosystems) or the relative quantification tool in LightCycler® 480 Software. The geNorm™ algorithm integrated into DataAssist™ was used to evaluate the stability of the candidate reference genes. The expression level and standard deviation for each gene was represented as the column charts using GraphPadPrism. All the candidate reference genes (B2m, Gapdh, Hprt, and Rplp1) displayed high expression stability, as determined by the geNorm tool [38], and therefore were used for the normalisation of the expression data. Data are the mean ± standard deviation of two (C2C12) or three biological replicates, each analysed in two technical replicates. Results were analysed by ratio paired *t* test and differences were considered statistically significant when *P* <0.05.

Analyses of mRNA levels in BM-MSCs and ESCs included RNA isolation using the mirVana kit (Life Technologies) and then reverse transcription using Superscript (Life Technologies). The TaqMan assays (Life Technologies) and Master Mix (Life Technologies) were used to analyse the level of the genes CXCR4, CXCR7, and CD9 according to the PCR array manufacturer’s instructions. Hprt1 was used as the reference gene. All reactions were performed in triplicates. The conditions of quantitative RT-PCR were as follows: reverse transcription, 25°C for 10 minutes, 42°C for 60 minutes, and 85°C for 5 minutes; quantitative PCR, template denaturation, 95°C for 10 minutes, and 45 cycles of 95°C for 15 seconds and 60°C for 60 seconds. The collected data were analysed using LightCycler 96SW 1.1 software (Roche Applied Sciences). Data are the mean ± standard deviation of three biological replicates, each analysed in two technical replicates. Results were analysed by paired *t* test and differences were considered statistically significant when *P* <0.05.

### Immunocytochemistry

Selected antigens were immunolocalised in sections of regenerating muscles, as well as in *in vitro* cultured cells. Cells cultured were fixed with 3% paraformaldehyde for 10 minutes. Muscle cryosections were hydrated in PBS, fixed in 3% paraformaldehyde and washed with PBS. Next, sections or cells were permeabilised with 0.1% Triton X-100/PBS (Sigma-Aldrich), and incubated with 0.25% glycine (Sigma-Aldrich). Nonspecific binding of antibodies was blocked with 3% bovine serum albumin (Sigma-Aldrich) with 2% donkey serum (Sigma-Aldrich) for 1 hour. Samples were then incubated with primary antibodies diluted 1:100 in 3% bovine serum albumin overnight, washed with PBS, and incubated at room temperature with secondary antibodies diluted 1:200 in 3% bovine serum albumin for 1.5 hours. After washing with PBS, cell nuclei were visualised by incubation with DraQ5 (Biostatus Limited) diluted 1:1,000 in PBS for 10 minutes. Specimens were mounted with Fluorescent Mounting Medium (Dako Cytomation, Glostrup, Denmark). After the procedure was completed samples were analysed using the confocal microscope Axiovert 100 M (Zeiss) and LSM 510 software. The same image acquisition settings were used for all comparisons. The following primary antibodies were used: rabbit polyclonal anti-skeletal myosin (M7523; Sigma-Aldrich), rabbit polyclonal anti-β-galactosidase (ab12081; Abcam), mouse monoclonal anti-integrin alpha3 (sc-7019; Santa Cruz Biotechnology, Santa Cruz, CA, USA), rabbit polyclonal anti-integrin beta1 (sc-9936; Santa Cruz), rabbit polyclonal anti-ADAM12 (ab39155; Abcam), rabbit polyclonal anti-CD9 (C9993; Sigma-Aldrich), rabbit polyclonal anti-CXCR4 (ab2074; Abcam), rabbit polyclonal anti-CXCR7 (ab117836; Abcam), goat polyclonal anti-CD81 (sc-7102; Santa Cruz), mouse monoclonal anti-M-cadherin (ab78090; Abcam), and rabbit polyclonal anti-VCAM-1 (sc-8304; Santa Cruz). Secondary antibodies directed against mouse or rabbit primary antibodies conjugated with Alexa488, Alexa594, and Alexa633 were used (A21202, A11059, A21206, A11034, A11080, A21203, A11037, A21071, A21082, A21063; Life Technologies). Appropriate controls of secondary antibodies were performed.

### Western blotting

Fifty micrograms of total protein lysate were denatured by boiling in Laemmli buffer, separated using SDS-PAGE electrophoresis, and transferred to polyvinylidene fluoride membranes (Roche Applied Science). The membranes were washed, blocked with 5% Blotto (BioRad, Bio-Rad, Hercules, CA, USA) and Tris-buffered saline for 1 hour, and incubated at 4°C with primary antibodies diluted 1:2,000 in 5% Blotto (BioRad) and Tris-buffered saline overnight, followed by secondary antibodies diluted 1:20,000 for 2 hours. Next, protein bands were visualised with SuperSignal West Pico Chemiluminescent Substrate (Thermo Fisher Scientific, Langenselbold, Germany) and exposed to chemiluminescence positive film (Amersham Hyperfilm ECL; GE Healthcare, Little Chalfont, Buckinghamshire, United Kingdom). The obtained results were analysed with GelDoc2000 using Quantity One software (BioRad). Primary antibodies used were mouse monoclonal anti-integrin alpha3 (sc-7019; Santa Cruz), rabbit polyclonal anti-integrin beta1 (sc-9936; Santa Cruz), rabbit polyclonal anti-ADAM12 (ab39155; Abcam), rabbit polyclonal anti-CD9 (C9993; Sigma-Aldrich), goat polyclonal anti-CD81 (sc-7102; Santa Cruz), rabbit polyclonal anti-CXCR4 (ab2074; Abcam), rabbit polyclonal anti-CXCR7 (ab117836; Abcam), rabbit polyclonal anti-M-cadherin (sc-10734; Santa Cruz), rabbit polyclonal anti-VCAM-1 (sc-8304; Santa Cruz), and mouse monoclonal anti-tubulin (T5168; Sigma-Aldrich). Secondary antibodies used were peroxidase-conjugate rabbit anti-mouse (A9044; Sigma-Aldrich), peroxidase-conjugate rabbit anti-goat (A5420; Sigma-Aldrich), and peroxidase-conjugate goat anti-rabbit (A9169; Sigma-Aldrich). Three independent experiments were performed.

## Results

### Sdf-1 treatment changes expression of adhesion proteins during myoblast differentiation *in vitro* and *in vivo* in regenerating muscle

In our previous studies we evidenced that Sdf-1 improved muscle regeneration, stem cell mobilisation, and myoblast migration [30]. Since adhesion proteins play a crucial role in the myogenic processes we decided to focus on the possible link between Sdf-1 and those proteins engaged in myoblast migration and differentiation. To verify the existence of such a link we first focused on skeletal muscle regeneration.

**Figure 1:**
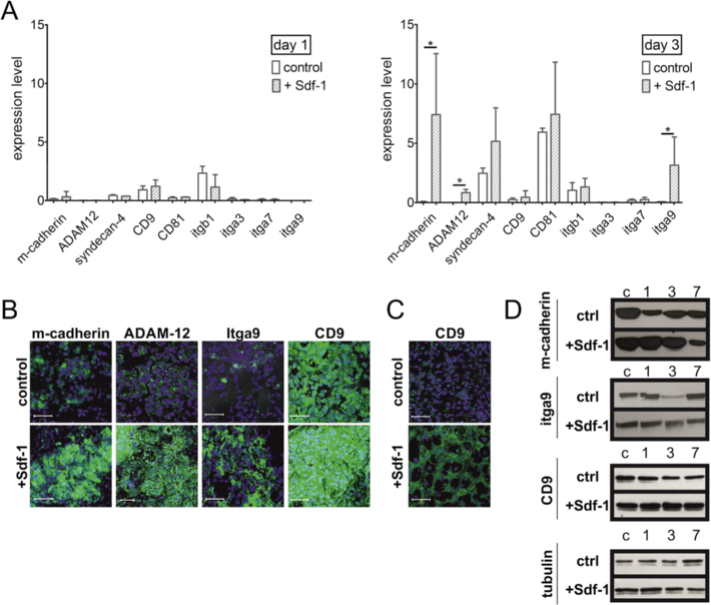
**Sdf-1 impact on the expression of adhesion proteins in regenerating rat soleus muscle. (A)** Quantitative RT-PCR analysis of mRNAs encoding m-cadherin, ADAM-12, syndecan-4, CD9, CD81, integrin β1 (itgb1), integrin α3 (itga3), integrin α7 (itga7), and integrin α9 (itga9) in control and Sdf-1-treated muscles (Sdf-1) at days 1 and 3 of regeneration. **(B)** Immunolocalisation of m-cadherin, ADAM-12, itga9, and CD9 in control and Sdf-1-treated muscles at day 3 of regeneration. Bar = 50 μm. **(C)** Immunolocalisation of CD9 in control and Sdf-1-treated muscles at day 7 of regeneration. **(D)** Level of m-cadherin, itga9, and CD9 protein during control (ctrl) and Sdf-1-treated muscle regeneration at days 1, 3, and 7 (C – intact muscle). *P <0.05. Error bars indicate standard deviation.

To follow the impact of Sdf-1 on regeneration, soleus muscles of WAG rats were injected with Sdf-1 (100 ng per muscle) after the muscle injury. Next, we analysed nontreated (control) and Sdf-1-treated muscles at days 1 and 3 of regeneration (Figure 1). Activated satellite cells start to proliferate (day 1), differentiate into myoblasts (day 3) that fuse (day 7) to form myotubes, and reconstruct damaged myofibres. The levels of mRNAs encoding adhesion proteins (that is, i.e. m-cadherin, ADAM-12, syndecan-4, CD9, CD81, integrin beta1, alpha3, alpha7, and alpha9) were compared between control and Sdf-1-treated muscles, at days 1 and 3 of regeneration. At day 3, Sdf-1 significantly increased expression of m-cadherin, ADAM-12, and integrin alpha9 at the mRNA level (Figure 1A). Changes in mRNA levels was readily translated to the levels of m-cadherin, ADAM-12, and integrin alpha9 proteins, which dramatically increased in mononucleated cells present within the regenerating muscle (day 3), as shown by immunolocalisation (Figure 1B). We did not observe significant changes in the mRNAs encoding other analysed factors; that is, syndecan-4, CD9, CD81, integrin beta1, alpha3, and alpha7. However, immunolocalisation revealed that Sdf-1 impacted one of the tetraspanins (that is, CD9). Immunolocalisation of CD9 showed that this tetraspanin was present in mononucleated cells both in control and Sdf-1-treated muscles at day 3 of regeneration of the soleus muscle (Figure 1B). At day 7 of regeneration, CD9 was still detectable in mononucleated cells and rarely in newly formed myofibres in control muscles (Figure 1C). However, in Sdf-1-treated muscles this protein was detectable in mononucleated cells and significantly in the cell membranes of newly formed myofibres (Figure 1C). Western blotting also showed the changes at the protein level after Sdf-1 treatment (Figure 1D). The level of m-cadherin, itga9, and CD9 increased at days 1 and 3 of muscle regeneration in response to Sdf-1 treatment. The changes in CD9 level were also detectable at day 7 (Figure 1D). Importantly, as we showed previously, Sdf-1 did not change the number of rat satellite cell-derived myoblasts during *in vitro* culture, implying that it did not impact the proliferation rate [30].

**Figure 2:**
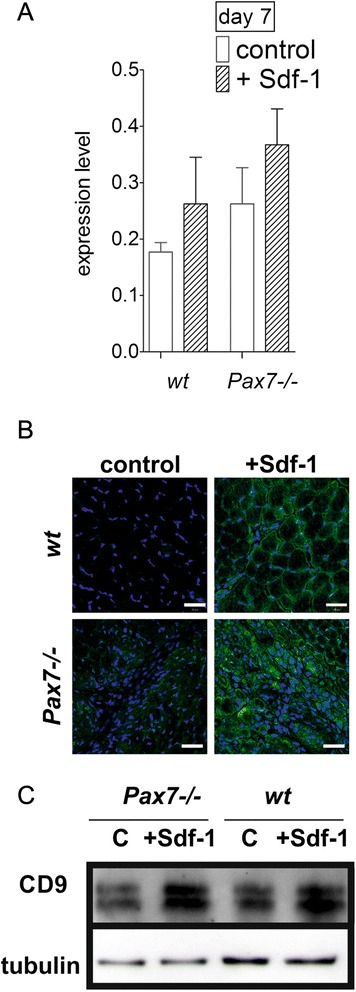
**CD9 in control and injected with Sdf-1 skeletal muscles of wild type and** ***Pax7*** ^***–/–***^ **mice. (A)** Quantitative RT-PCR analysis of mRNA encoding CD9 in wild type (*wt*) and *Pax7* ^*–/–*^ mice muscles at day 7 of regeneration. **(B)** Immunolocalisation of CD9 in *wt* and *Pax7* ^*–/–*^ mouse muscles at day 7 of regeneration. Nuclei, blue; adhesion proteins, green. Bar = 30 μm. **(C)** Level of CD9 protein during control (C) and Sdf-1-treated muscle regeneration of *wt* and *Pax7* ^*–/–*^ mice at day 7. Error bars indicate standard deviation.

To determine whether Sdf-1 increases CD9 expression in satellite cell-derived myoblasts or impacts on cells migrating to the injured muscle, we decided to focus on *Pax7*^*–/–*^ mice. Previous analyses of this mouse model did not show any abnormalities in embryonic myogenesis [39]. However, postnatal development in *Pax7*^*–/–*^ mice is associated with a dramatic decrease in the population of satellite cells, which causes the muscle growth retardation [40]. As a result these mice are significantly smaller than wild-type (*wt*) mice, have difficulty in moving, and usually die within 3 weeks of age. Analyses of *Pax7*^*–/–*^ muscles give us a unique opportunity to answer the question about the identity of cells upregulating CD9 within the injured muscle; that is, we were able to test whether Sdf-1 treatment impacted on the resident satellite cells (absent in *Pax7*^*–/–*^ mice) or the cells that were infiltrating injured muscle. Again, Sdf-1 treatment increased the CD9 mRNA level only slightly (Figure 2A). However, the level of CD9 protein was higher in *Pax7*^*–/–*^ and *wt* mice muscles as showed by immunocytochemistry and western blotting (Figure 2B,C). CD9 protein exists in three forms with molecular masses between 22 and 27 kDa, and thus two CD9 bands were detected by western blot. Summarising, we proved that Sdf-1 injected into the muscle upregulated the CD9 level also in cells other than the satellite cells that either are already present within or infiltrate regenerating muscle. To answer the question of whether Sdf-1 also acts at satellite cells, we turn to the *in vitro* system.

**Figure 3:**
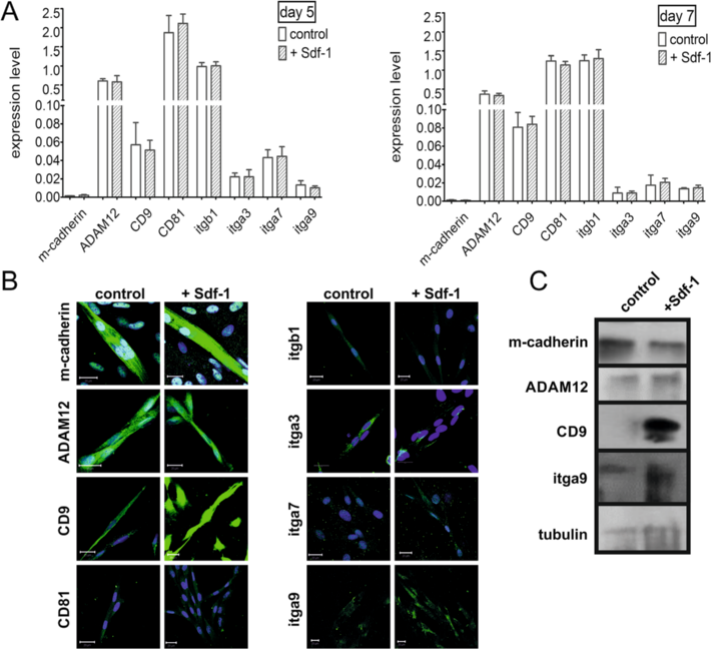
**Sdf-1 impacts on the expression of adhesion proteins (m-cadherin, ADAM-12, syndecan-4, CD9, CD81, integrin β1, integrin α3, integrin α7, integrin α9) in differentiating rat satellite cell-derived myoblasts. (A)** Level of mRNA at days 5 and 7 of control (C) and Sdf-1-treated myoblast (Sdf-1) differentiation. **(B)** Immunolocalisation of adhesion proteins in control and Sdf-1-treated cells. Nuclei, blue; adhesion proteins, green. Bar = 20 μm. **(C)** Level of adhesion proteins during control and Sdf-1-treated myoblast differentiation at day 7. itgb1, integrin β1; itga3, integrin α3; itga7, integrin α7; itga9, integrin α9. *P <0.05. Error bars indicate standard deviation.

The notion that Sdf-1 treatment results in upregulation of CD9 in satellite cells was tested in *in vitro* experiments in which we took advantage of primary rat satellite cell-derived myoblasts. At day 5 of culture, satellite cell-derived myoblasts start to proliferate and then, at day 7, fuse to form multinucleated myotubes. We did not observe any significant differences in m-cadherin, ADAM-12, syndecan-4, CD9, CD81, integrin beta1, alpha3, alpha7, and alpha9 mRNA levels between control and Sdf-1-treated cells at day 5 (nondifferentiated cells) and day 7 of culture (fusing cells) (Figure 3A). When assessing the levels of the proteins by immunolocalisation and western blotting we observed a spectacular increase only in the case of CD9 (Figure 3B,C).

### Downregulation of Sdf-1 receptor (CXCR4) affects CD9 expression in C2C12 myoblasts

**Figure 4:**
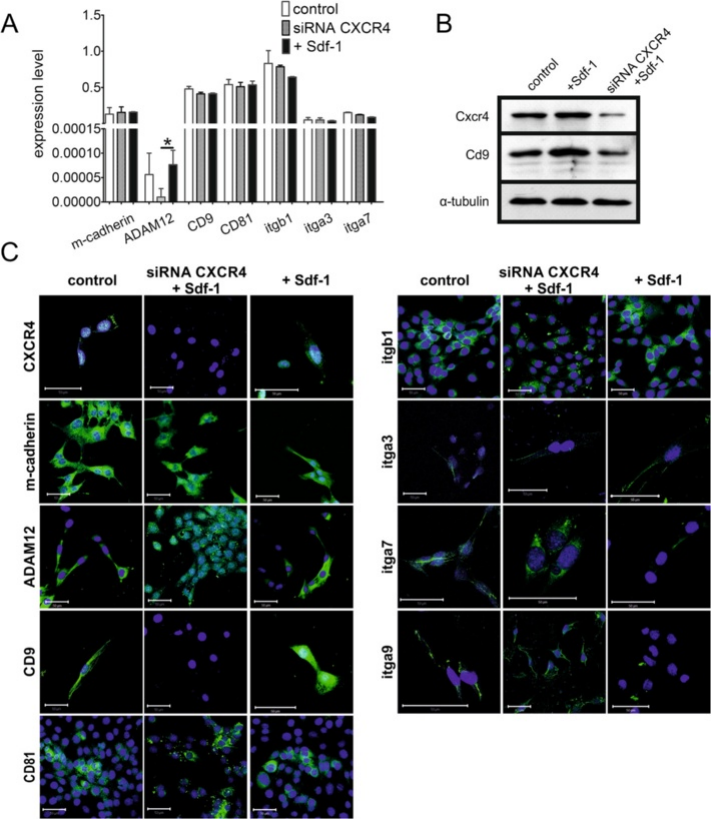
**Level of CXCR4 and adhesion proteins in C2C12 myoblasts. (A)** Quantitative RT-PCR analysis of mRNA encoding m-cadherin, ADAM-12, syndecan-4, CD9, CD81, integrin β1 (itgb1), integrin α3 (itga3), integrin α7 (itga7), and integrin α9 (itga9) in control cells, treated with Sdf-1, and transfected with CXCR4 siRNA (siRNA). **(B)** Western blotting analysis of CXCR4, CD9, and tubulin in control, Sdf-1-treated (Sdf-1) and transfected with CXCR4 siRNA (siRNA) C2C12 myoblasts. **(C)** Immunolocalisation of CXCR4 and adhesion protein in control, treated with Sdf-1, and transfected with CXCR4 siRNA (siRNA) C2C12 myoblasts. Nuclei, blue; adhesion proteins, green. Bar = 50 μm. CXCR, CXC chemokine receptor; siRNA, small interfering RNA. *P <0.05. Error bars indicate standard deviation.

To further dissect the Sdf-1 impact on myoblast differentiation we decided to manipulate the levels of its receptor; that is, CXCR4. In these experiments we used mouse C2C12 myoblasts. The rationale behind the choice of these cells is based on the fact that this cell line not only serves as a standard in the studies on myoblast differentiation, but also is easy to manipulate and transfect in *in vitro* culture. Expression of CXCR4 was downregulated with specific siRNA. Forty-eight hours following transfection with siRNA the level of CXCR4 mRNA decreased to 42.72 ± 2.39%, as compared with cells transfected with control siRNA cells. In addition, the level of CXCR7 mRNA, which was shown to be involved in the Sdf-1 and interferon-inducible T-cell chemoattractant signalling pathway [41], also slightly decreased to 77.14 ± 8.39%. Downregulation of CXCR4 did not change significantly the levels of mRNAs encoding adhesion proteins; that is, m-cadherin, ADAM-12, syndecan-4, CD9, CD81, integrin beta1, alpha3, and alpha7 (Figure 4A). Moreover, Sdf-1 treatment did not impact on the levels of analysed mRNAs (Figure 4A). However, the difference in ADAM12 mRNA level after silencing CXCR4 expression and Sdf-1 treatment was statistically significant (*P* = 0.046), but it was not statistically significant when compared with control. Next, the expression of itga9 mRNA was very low and changed neither after Sdf-1 treatment nor after downregulation of CXCR4 (data not shown). The downregulation of CXCR4 level was translated to the protein level (Figure 4B). Moreover, the level of CD9 protein increased after Sdf-1 treatment and decreased in response to CXCR4 downregulation (Figure 4B). Immunolocalisation again proved that CXCR4 protein was not detectable in siRNA transfected cells (Figure 4C). We also did not notice changes in the localisation and levels of m-cadherin, ADAM-12, syndecan-4, CD81, integrin beta1, alpha3, alpha7, and alpha9 (Figure 4C). Importantly, CXCR4 silencing abolished Sdf-1 induced CD9 upregulation (Figure 4C).

### Sdf-1 upregulates CD9 expression in bone marrow-derived stem cells and embryonic stem cells

Our next question was whether Sdf-1 affected CD9 expression in stem cells other than satellite cells or cells infiltrating the injured skeletal muscle. To address this issue we decided to analyse two standard stem cell types of different origin: multipotent adult BM-MSCs and pluripotent ESCs. Both types of cells are extensively studied as a source of cells that could be used in therapy.

**Figure 5:**
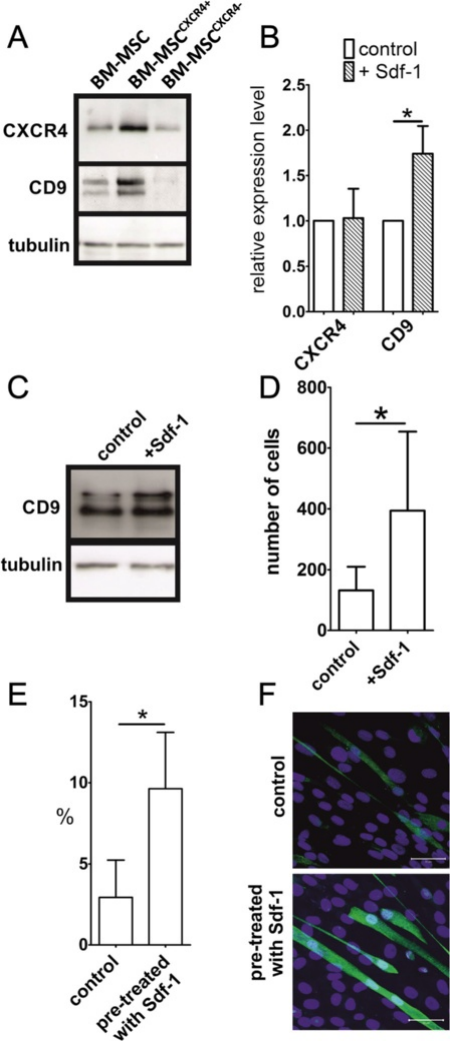
**Sdf-1 impact on bone marrow mesenchymal stem cells. (A)** Western blotting analysis of CXCR4, CD9, and tubulin in the whole population of bone marrow-derived mesenchymal stem cells (BM-MSCs) as well as of CXCR4^+^ and CXCR4^–^ BM-MSCs fractions fractions. **(B)** Quantitative RT-PCR analysis of CXCR4 and CD9 mRNA in BM-MSCs in control and Sdf-1-treated BM-MSCs. **(C)** Western blotting analysis of CD9 and tubulin in control and Sdf-1-treated (Sdf-1) BM-MSCs. **(D)** Migration of BM-MSCs in Sdf-1 gradient. The number of cells that migrated from the inserts was counted. **(E)** Percent of hybrid myotubes formed in co-culture of C2C12 myoblasts and control or Sdf-1 pretreated BM-MSCs. **(F)** Co-culture of C2C12 myoblasts and control or Sdf-1 pretreated BM-MSCs (green, localisation of β-galactosidase; blue, nuclei). Bar = 50 μm. CXCR, CXC chemokine receptor. *P <0.05. Error bars indicate standard deviation.

**Figure 6:**
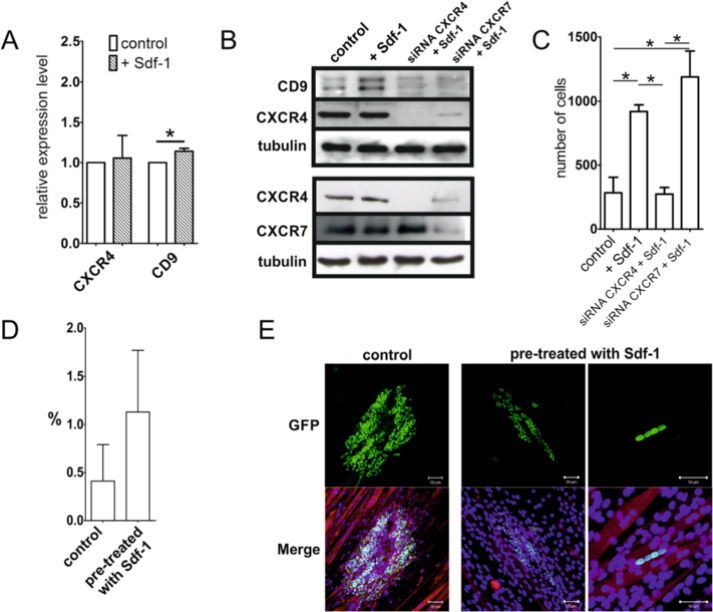
**Sdf-1 impact on embryonic stem cells. (A)** Quantitative RT-PCR analysis of CXCR4 and CD9 mRNA in control and Sdf-1-treated embryonic stem cells (ESCs). **(B)** Western blotting analysis of CXCR4, CXCR7, CD9, and tubulin in control, Sdf-1-treated (Sdf-1), and either CXCR4 (siRNA CXCR4) or CXCR7 siRNA-treated (siRNA CXCR7) ESCs. **(C)** Migration of control or transfected with CXCR4 or CXCR7 siRNA ESCs in response to Sdf-1 gradient. **(D)** Proportion of hybrid myotubes formed in co-culture of C2C12 myoblasts and control or Sdf-1 pretreated ESCs. **(E)** Co-culture of C2C12 myoblasts (red) and control or Sdf-1 pretreated ESCs (green); nuclei, blue. Bar = 50 μm. CXCR, CXC chemokine receptor. *P <0.05. Error bars indicate standard deviation.

Cells isolated from mouse bone marrow were separated using a magnetic column and the fraction of CXCR4-positive cells (BM-MSCs^CXCR4+^) – that is, only the cells able to react to Sdf-1 – were analysed. We showed that the level of CXCR4 protein is higher in BM-MSCs^CXCR4+^ than in BM-MSCs^CXCR4–^ or the whole population of BM-MSCs (Figure 5A). Importantly, Sdf-1 treatment lead to the significant increase of CD9 mRNA and protein levels in BM-MSCs^CXCR4+^ (Figure 5B,C). CD9 protein exists in three forms with molecular masses between 22 and 27 kDa, and thus two CD9 bands were detected by western blot. Next, we tested the impact of Sdf-1 on ESCs, control and transfected with siRNA against CXCR4 or CXCR7. The mRNA and protein level of CXCR4 did not change after Sdf-1 treatment and the level of CXCR4 protein was significantly downregulated in cells transfected with CXCR4 siRNA (Figure 6A,B). Notably, in the response to Sdf-1, ESCs also upregulated CD9 at the mRNA and protein levels (Figure 6A,B). Silencing of CXCR4 lead to the downregulation of CD9 protein in ESCs (Figure 6B). Downregulation of CXCR4 did not change the protein level of the second Sdf-1 receptor; that is, CXCR7 (Figure 6B). We also decided to silence expression of CXCR7 and observed that this only slightly reduced the CD9 protein level (Figure 6B). However, silencing of CXCR7 expression was connected with lower CXCR4 expression. We thus concluded that observed lower CD9 protein expression could be the result of CXCR4 downregulation.

### Stem cells treated with Sdf-1 migrate and fuse with myoblast more effectively than control cells

Having found that Sdf-1, acting via CXCR4, upregulates CD9 in myoblasts, cells infiltrating injured muscle, and in such stem cells as BM-MSCs and ESCs, we decided to assess whether this phenomenon contributes to the improvement of skeletal muscle regeneration. We used *in vitro* systems allowing assessment of the cell migration ratio and the myogenic potential of analysed cells.

First, the migration of BM-MSCs and ESCs in response to Sdf-1 was analysed. Using the transwell migration system we showed that the number of BM-MSCs which migrated in response to Sdf-1 increased 3.0 times (Figure 5D). The number of ESCs that migrated increased 3.25 times in the presence of Sdf-1 (Figure 6C). Silencing of CXCR4, but not CXCR7, expression lead to the decrease of ESC migration in response to Sdf-1 treatment (Figure 6C). Next, we analysed how Sdf-1 impacts on the myogenic potential of BM-MSCs or ESCs. To this point, we co-cultured cells pretreated with Sdf-1 with differentiating C2C12 myoblasts. Analysis of co-culture of BM-MSCs with C2C12 cells revealed that they were able to form 2.93 ± 2.3% of hybrid myotubes; that is, tubules formed as a result of fusion between tested stem cells and C2C12 myoblasts. Sdf-1 pretreatment increased this number to 9.63 ± 3.5% (Figure 5E,F). Control ESCs were able to form 0.41 ± 0.38% of hybrid myotubes. In response to Sdf-1 pretreatment, the number of hybrid myotubes increased to 1.13 ± 0.64% (Figure 6D,E). As we have shown previously, ESCs very rarely fuse with myoblasts [42]. The observed increase in the fusion index after Sdf-1 treatment was thus an interesting result. Taken together, our results indicate that Sdf-1 upregulated CD9 expression in a CXCR4-dependent, but not a CXCR7-dependent, manner, induced stem cell migration, and increased myogenic potential of analysed stem cells.

## Discussion

Previously, we showed that Sdf-1 improved regeneration of injured skeletal muscles by inducing stem cell mobilisation to injured muscle and also increasing myoblast migration via matrix metalloproteinases MMP2 and MMP9 [30]. However, Sdf-1 did not change the expression of myogenic regulatory factors either *in vivo* or *in vitro* [30]. Next, many lines of evidence, including our own studies, showed that adhesion proteins play a crucial and indispensable role in skeletal muscle regeneration [32,33,43,44]. Thus, in the current work we tested whether and how Sdf-1 affects expression of adhesion proteins engaged in myoblast migration and differentiation. We found that the levels of adhesion proteins increased in Sdf-1-treated muscles but not in *in vitro* cultured myoblasts (primary culture or cell line). This led us to the suggestion that *in vivo* the increase of m-cadherin, integrin alpha9, and ADAM12 could occur in cells other than myoblasts engaged in the regeneration of skeletal muscle. Nevertheless, our most important observation was that Sdf-1 induced upregulation of CD9 *in vivo* during *wt* and *Pax7*^*–/–*^ muscle regeneration and in *in vitro* cultured myoblasts and such stem cells as BMSCs and ESCs.

CD9 is a 21 to 24 kDa surface molecule that belongs to the tetraspanins, a family of four-transmembrane domain proteins associated with integrin receptors, which was described as motility-related factor engaged in migration of multiple cancer cell lines [45]. CD9 was also shown to be associated with such integrins as α3β1, α4β1, α5β1, α6β1, α6β4, and αIIbβ3 [45]. Interactions of CD9 with integrins led to changes in their conformation and activation, which results in the modulation of integrin-dependent signalling pathways [46]. Moreover, CD9 is directly associated with EWI-2 and CD9P-1 (also known as EWI-F or FPRP, member of the immunoglobulin superfamily), epidermal growth factor receptor, and discodin domain receptor DDR1 [47-49]. Additionally, the tetraspanin network modulates membrane-type 1 matrix metalloproteinase cell surface localisation and is able to induce expression and also activate MMP2 [50,51]. By impacting at matrix metalloproteinases, CD9 may regulate not only cell migration but also tissue remodelling during embryonic development, angiogenesis, tumour invasion and metastasis, and also tissue regeneration. Importantly, CD9 was also shown to play a role in muscle fibre formation [52]. In 1999 Tachibana and Hemler documented that anti-CD9 antibodies inhibited fusion of mouse C2C12 myoblasts, without affecting muscle-specific protein expression such as myosin heavy chains, desmin, and actin [52]. In our previous study, we also showed that the complex of CD9 and integrin α3β1 plays a pivotal role during satellite cell-derived myoblast fusion and skeletal muscle regeneration [33]. Interestingly, β1-deficient myoblasts that were unable to fuse did not express CD9 [53]. Moreover, Charrin and coworkers showed that proper muscle regeneration required CD9 and CD81 function [54]. They demonstrated that mice lacking either CD9 or CD81, or both CD9 and CD81, were unable to properly regenerate their skeletal muscles. During reconstruction of CD9 and CD81-deficient muscle, myoblasts formed giant dystrophic myofibres. Also *in vitro* absence of both CD9 and CD81 led to hyperfusion of myoblasts. Myoblasts lacking either CD9 or CD81 fused *in vitro* normally.

If a lack of CD9 decreases cell fusion, then its upregulation should have the opposite effect. Indeed, human rhabdomyosarcoma-derived myoblasts overexpressing CD9 formed approximately fourfold more syncytia than control cells [52]. In the current study we showed that Sdf-1 seems to be a perfect trigger leading to the increase in the CD9 proteins levels that promotes skeletal muscle regeneration via induction of stem cell migration and fusion with myoblasts. First, we noticed that Sdf-1 treatment results in upregulation of CD9 in myoblasts in a CXCR4-dependent way. Next, using *Pax7*^*–/–*^ mice, we showed that Sdf-1 treatment also increased CD9 expression in cells other than satellite cells and differentiating myoblasts; that is, stem cells that infiltrate regenerating muscles. Our *in vitro* studies focusing on BM-MSCs and ESCs proved that stem cells are prone to Sdf-1/CXCR4-dependent CD9 induction, which leads to their increased migration and ability to fuse with myoblasts. Thus, we suggest that preconditioning of stem cells with Sdf-1 could be an alternative approach to optimise stem cell migration and engraftment after their injection into injured skeletal muscle. Presently, the major limitation causing the failure of clinical trials is the lack of specific homing of cells transplanted into injured tissue [55]. Some evidence shows that Sdf-1 treatment could be a strategy to improve the therapeutic potential of stem cells [56]. Sdf-1 treatment of endothelial progenitor cells improved their migration and adhesion to activated endothelium [57]. Sdf-1-treated endothelial progenitor cells from human umbilical cord or cord blood upregulated expression of integrins (α4 and αM) and MMP2 secretion [57]. Moreover, Sdf-1-treated mesangioblasts migrated more effectively *in vitro*, and *in vivo* efficiently engrafted mouse dystrophic muscles improving the reconstruction of muscle fibres [58]. BM-MSCs preconditioning with Sdf-1 increased cell viability, proliferation, and vascular endothelial growth factor secretion *in vitro* [59]. Sdf-1 was also shown to promote homing and proliferation of transplanted MSCs into infarcted myocardium [59]. Importantly, rat hearts transplanted with Sdf-1-pretreated MSCs showed significant neoangionesis in the ischaemic area [59].

The therapeutic potential of MSCs such as BM-MSCs is extensively explored. MSCs can be easily isolated from adult tissues and cultured *in vitro*. Notably, these cells exhibit no significant immunogenicity [60,61] and are able to differentiate into various cell types, producing cytokines and growth factors characterised by anti-apoptotic, anti-inflammatory, and pro-angiogenic properties [62]. MSCs are also able to effectively follow the myogenic programme [17]. On the other hand, ESCs that are characterised by the potential to differentiate *in vivo* into any given cell type fail to efficiently produce some cell types *in vitro*. Myogenic differentiation of ESCs does not occur spontaneously even in embryonic bodies that mimic spatiotemporally early embryonic development [63,64]. As was shown by Darabi and coworkers, ESC overexpression of *Pax3* or *Pax7* can effectively drive the cells into a myogenic programme [25,26,65]. Other *in vitro* methods, such as culture conditions [66] or various chemical treatments [67], are far less effective. Here, we showed that upregulation of CD9, as the result of Sdf-1 pretreatment, leads to the increase in ability to migrate and fuse with myoblasts of these two stem cell lines; that is, BM-MSCs and ESCs.

The mechanism of CD9 expression is particularly interesting. It is known that CD9 mRNA exists in two major RNA species differing only in the length of their 5′ untranslated region [68]. Efficient mRNA translation depends, among other factors, on the supportive RNA folding of the 5′ untranslated region; that is, the region which contains the initiation codons. The long and short forms of the 5′ untranslated region of CD9 mRNA have different stability. The long 5′ untranslated region is characterised by a complex secondary structure comprising a stable stem-loop. A shift from shorter to longer 5′ untranslated regions influences the CD9 protein level. Thus, not only a reduction or increase in the absolute quantity of CD9 mRNA can reduce or increase the level of CD9 protein. Moreover, the mechanism of CD9 upregulation is concerned with some suggestions coming from the study, which showed in human CD34^+^ cells isolated from cord blood that Sdf-1 acting through CXCR4 induced expression of CD9 via G-proteins and kinases they activate – protein kinase C, phospholipase C, extracellular signal-regulated kinase, and Janus kinase 2 signals [69]. Furthermore, pretreatment of human CD34^+^ cells with anti-CD9 antibody significantly impaired their spleen and bone marrow homing [69].

## Conclusions

**Figure 7:**
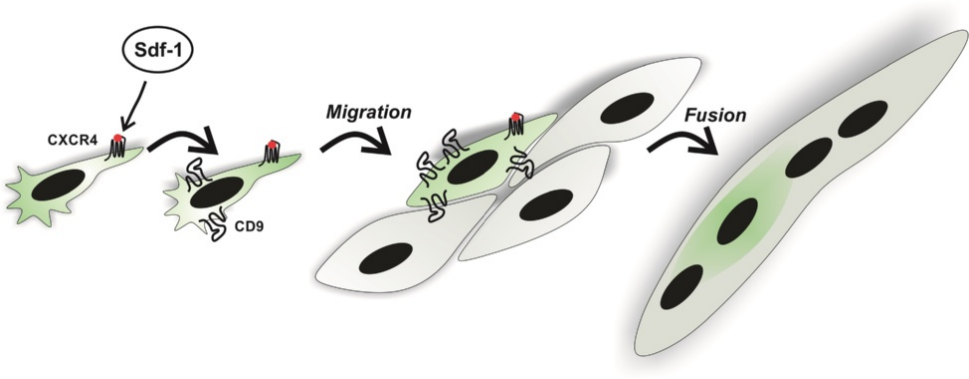
**Myogenic potential of stem cells is not sufficient to apply them in damaged skeletal muscle therapy.** Induction of CD9 expression in a Sdf-1 CXCR4-dependent way leads to increased ability of stem cell lines to migrate and fuse with myoblasts. CXCR, CXC chemokine receptor.

The myogenic potential of stem cells is not sufficient to apply the cells in damaged skeletal muscle therapy. Here, we have shown that Sdf-1, through the CXCR4 receptor, induced expression of tetraspanin CD9 in satellite cell-derived myoblasts, ESCs, and BM-MSCs. We showed that upregulation of CD9 led to an increase in the ability of stem cells lines to migrate and fuse with myoblasts (Figure 7). Induction of CD9 could thus increase the therapeutic potential of stem cells.

## Abbreviations

AB: antibiotics
BM-MSC: bone marrow-derived mesenchymal stem cell
CXCR: CXC chemokine receptor
DMEM: Dulbecco’s modified Eagle’s medium
ESC: embryonic stem cell
FBS: foetal bovine serum
MSC: mesenchymal stem cell
Sdf-1: stromal-derived factor-1
siRNA: small interfering RNA
